# Infections due to Carbapenem Resistant Enterobacteriaceae among Saudi Arabian Hospitalized Patients: A Matched Case-Control Study

**DOI:** 10.1155/2016/3961684

**Published:** 2016-04-06

**Authors:** M. A. Garbati, H. Sakkijha, A. Abushaheen

**Affiliations:** ^1^Section of Infectious Diseases, Medical Specialties Department, King Fahad Medical City, P.O. Box 59046, Riyadh 11525, Saudi Arabia; ^2^Pulmonary and Critical Care Medicine Department, King Fahad Medical City, P.O. Box 59046, Riyadh 11525, Saudi Arabia; ^3^Scientific Research and Publication Center, King Fahad Medical City, P.O. Box 59046, Riyadh 11525, Saudi Arabia

## Abstract

*Background*. We conducted this case-control study to determine the risk factors and treatment outcome of infections due to carbapenem resistant Enterobacteriaceae in our institution.* Methods*. This is a matched case-control study of patients with infection due to carbapenem resistant Enterobacteriaceae (CRE) and carbapenem susceptible Enterobacteriaceae (CSE), from Riyadh, Saudi Arabia, between March 2012 and December 2013.* Results*. During this period, 29 cases and 58 controls were studied. The mean ages of the cases (55.4 years) and controls (54.7 years) were similar (*p* = 0.065). Cases had higher mean Charlson comorbidity index (CCI) (3.1) than controls (1.9), *p* = 0.026. Several factors contributed to infection among the studied population. Prior uses of piperacillin-tazobactam, a carbapenem, a quinolone, and metronidazole were significantly associated with CRE infections. Nine of the cases died compared with 7 of the controls, *p* = 0.031. Mortality was associated with advanced age, the presence of comorbidities, ICU stay, and receipt of invasive procedures.* Conclusions*. Infections due to CRE resulted in a significantly increased mortality. Combination antibiotic therapy was associated with reduced mortality. Properly designed randomized controlled studies are required to better characterize these findings.

## 1. Introduction

Carbapenems are antibiotics of last resort used to treat severe infections due to Gram-negative rods (GNR), such as those caused by extended spectrum *β*-lactamase- (ESBL-) producing organisms [1]. Unfortunately, resistance to these life-saving drugs has increasingly been reported among the most clinically relevant bacteria [2, 3], selective pressure being the major determinant [4]. Resistance to carbapenems is predominantly conferred by carbapenemases, such as oxacillinase- (OXA-) type enzymes and metallo-*β*-lactamases (MBLs) of Imipenemase (IMP), New Delhi metallo-*β*-lactamases (NDM) and Verona, Italy, metallo-*β*-lactamases (VIM) types, and serine carbapenemases of Klebsiella pneumoniae (KPC) type [3].

During the last decade increased prevalence of infections due to carbapenem resistant Enterobacteriaceae (CRE) has been reported [5]. Significant morbidity and mortality have been attributed to infections due to these isolates, especially bacteremia with attributable mortality rates of up to 50% compared with carbapenem susceptible Enterobacteriaceae (CSE) [6–9]. This is mainly related to delays in providing effective therapy. Previous case-control studies have identified various risk factors associated with carbapenem resistant* Klebsiella pneumoniae* (CRKP) infections [10, 11] that included antibiotic exposure, intensive care unit (ICU) stay, and poor functional status. Recently, Hussein et al. [12] reported that prior exposure to any antibiotic increases the chances of infection with CRE (OR 3.3 (95% CI 1.3–8.5), *p* = 0.012).

The optimal treatment of infections due to CRE is yet unknown. With few new antimicrobials under development, clinicians have resorted to using older, previously discarded antimicrobials, such as colistin and tigecycline, alone or in combination with carbapenems, aminoglycosides, and fosfomycin, to treat these infections [13, 14]. This approach has recently led to the emergence of organisms with resistance to antibiotics from all known classes, including the polymyxins [15]. Current clinical evidence for treatment guidance is limited and based on retrospective observational studies and case reports [15, 16]. A recent ICU outbreak of* Klebsiella oxytoca* infection revealed that monotherapy tended to be associated with higher mortality compared with combination therapy (60% versus 16.6%, *p* = 0.07) [17].

As observed, the threat of infections due to multidrug resistant Gram-negative bacteria (MDR GNB) is a growing concern across the Middle East but the risk factors for acquisition, as well as treatment outcomes, have not been previously characterized. Thus, the aim of this study was to identify the prevalence of infections due to CRE, in relation to the risk factors and outcome of treatment.

## 2. Methods

### 2.1. Study Site and Population

A matched case-control study was conducted on adult patients attending King Fahad Medical City (KFMC), Riyadh, between March 2012 and December 2013.

### 2.2. Subject Definitions and Study Protocol

#### 2.2.1. Cases

Patients were enrolled as cases if they had a new diagnosis of hospital-acquired infection with CRE.

#### 2.2.2. Controls

Patients were enrolled as controls if they had a new diagnosis of hospital-acquired infection with at least one culture positive for CSE. For each patient with CRE infection, we selected two controls matched for anatomic site of infection and the causative organism.

The study focused on the first episode of hospital-acquired infection even if recurrent infections occurred. All data were captured prospectively, and no additional tests were performed for the purpose of this study. Identification of cases for inclusion in the study was done consecutively and triggered from the microbiology laboratory where any isolate of CRE (mainly* Klebsiella pneumoniae*,* Escherichia coli*,* Enterobacter species*, and* Citrobacter species*) was flagged by the personnel who alerted the investigators. Controls were selected consecutively from a list of all CSE isolated in the microbiology laboratory of our center during the study period and their data were extracted prospectively. Due to the rarity of infection with some of these organisms, controls were selected without randomization. All identified eligible controls were screened for inclusion.

### 2.3. Microbiological Testing

Identification of infecting organisms (CRE or CSE) was performed using routine microbiological methods. Susceptibility testing for meropenem and imipenem was performed by both the disc diffusion method and an automated broth microdilution method (bioMerieux, Vitek II, Hazelwood, MO, USA), according to manufacturer's instructions. Isolates from clinical specimens during the study period were identified as carbapenem resistant when imipenem and/or meropenem resistance was documented. Antibiotic susceptibility was interpreted as per criteria published by The Clinical and Laboratory Standards Institute (CLSI) [18]. Susceptibility of tigecycline was determined by the use of minimum inhibitory concentration (MIC) break points approved by the US Food and Drug Administration (US-FDA) [19]. For colistin, break points proposed by the European Committee on Antimicrobial Susceptibility Testing (EUCAST) were used because relevant break points were not available from CLSI [20]. In this study we did not perform further tests to identify the production of specific types of carbapenemases or other mechanisms conferring carbapenem resistance.

### 2.4. Data Collection

The patient's location was identified and we collected the data prospectively using a structured data sheet. Information collected included age, sex, and patient source (home or another healthcare facility). Also, extracted were duration of current hospitalization, site of infection, treatment for the index infection, and the outcome. Severity of illness was assessed by the Charlson comorbidity index (CCI) [21], hospitalization within the previous three months, patient location at the time of infection, admission to ICU, antibiotic use within three months prior to the index admission, presence of a central venous catheter (CVC), urinary catheter, mechanical ventilation, and dialysis. The presence of comorbid conditions such as cardiovascular disease, lung disease, diabetes mellitus, solid tumors or hematological malignancy, liver disease, renal failure, and chemotherapy was also documented.

The microbiological data included the causative organisms isolated from the sites of infection, the date of isolation, and the* in vitro* susceptibilities of the organisms to various antibiotics, including colistin and tigecycline. All collected data were independently verified by two of the authors (MAG and AAS). For patients with more than one episode of infection only data from the first episode was collected and analyzed. These cases were followed up to determine treatment received and outcome. Exposure to various risk factors was taken into consideration in the analysis only if it had occurred prior to the acquisition of the infection. Prior antibiotic exposure was considered significant for analysis only if (i) that exposure had occurred within three months prior to the index hospitalization and (ii) the antibiotic had been administered for at least 72 hours.

### 2.5. Statistical Analysis

Data was described as mean ± SD and percentages. Least significant difference was measured at 95% CI. Intergroup comparison for metric variables was done by Student's *t*-test, whereas chi-square test and odds ratio were used for nonmetric variables. Binary logistic regression analysis for multivariate comparison and Kaplan-Meir Survival analysis predicted final outcome of the study. Analysis was performed by SPSS 22.0, JAVA stat, and MS Excel software.

### 2.6. Ethical Consideration

Written informed consent was obtained from all participants. The Ethics and Research Committee of KFMC approved the study.

## 3. Results

### 3.1. Study Population

Between March 2012 and December 2013, 29 cases of healthcare-associated infections due to CRE occurred. Fifty-eight control patients infected with CSE were consecutively selected in a ratio of 1 : 2 for cases and controls, matched for infecting organism and anatomic site of infection.

There were 62.1% and 55.2% males among the cases and controls (*p* = 0.540) with an age range of 15–94 years, mean 55.4 ± 3.8 (17–85 years) compared with 54.7 ± 2.6 (15–94 years) among the cases and controls, respectively, *p* = 0.065 (Table 1). Ninety-eight percent of the studied population were of Saudi extraction. The majority of the cases and controls were admitted from home (75% versus 81%). Cases were admitted into medical (31%) and surgical (24.1%) wards, while 44.8% went to the ICU, corresponding to 44.8%, 15.5%, and 39.7%, respectively, among the controls, *p* = 0.402. However, more cases had longer hospital stay than controls (*p* = 0.033), had more comorbidities (*p* = 0.002), had undergone more procedures (*p* < 0.001), and had higher CCI (*p* = 0.013) (Table 1). Among all the comorbid conditions studied, it is only renal disease requiring dialysis that was found to be independently associated with a CRE infection, 48.3% compared with (22.4%) controls (OR 3.23 (1.24–8.39) (*p* = 0.014)), Table 2. More cases received an antibiotic in the previous three months prior to the index admission, compared with controls (*p* < 0.001).

### 3.2. Type of Infection

Among the cases, the isolates included* Klebsiella pneumoniae* (15),* Escherichia coli* (7),* Enterobacter *sp. (6), and* Citrobacter *sp. (1) that, respectively, caused infections in the bloodstream (4), skin and soft tissue (7), urinary tract (7), and body fluids, pleural, pericardial, peritoneal, cerebrospinal, biliary, tracheal, and abscess contents and discharges from wounds (11), while the corresponding figures among the controls were 31, 13, 12, and 2 for the organisms and 7, 10, 17, and 24 for the sites, respectively. There were no statistically significant differences between the cases and controls with regard to the site of infection for blood (*p* = 1.000), tissue (*p* = 0.444), urine (*p* = 0.611), and body fluids (*p* = 0.757), similarly to the infecting organism (*p* = 0.998).

### 3.3. Risk Factors

By univariate analysis (Table 2) CRE infection was associated with duration of current hospitalization, antibiotic use in the previous three months, ICU stay, prior surgery, urinary catheterization, renal disease requiring dialysis, the use of any invasive procedure, and mechanical ventilation. Eighteen (62%) of the cases had been exposed to at least one antibiotic within the previous three months, compared with 11 (19%) of the controls (*p* < 0.001). On multivariate analysis, the duration of hospitalization, invasive procedures, and use of carbapenem were associated with infection with CRE. Not being in the ICU was protective against CRE infection (Table 3).

### 3.4. Outcome

**Figure 1:**
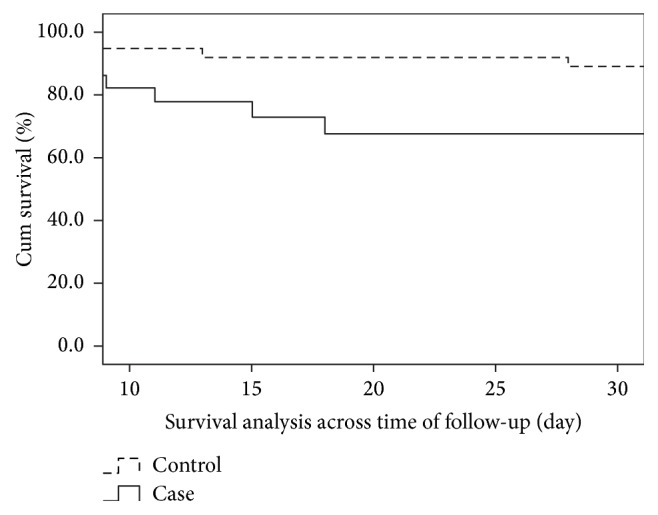
Survival curve using the Kaplan-Meier method for cases compared with controls. The cases (—) had a lower probability of survival than the controls (- - -) (*p* = 0.013).

Nine of 29 patients (31%) with CRE infection died compared with 7/58 (12.1%) of their matched controls (OR 3.28 (1.08–9.98), *p* = 0.031), with a nonsignificant higher mortality rate among males compared with females (13 (26.0%) versus 3 (8.1%) OR 3.98 (0.94–19.4), *p* = 0.064). The results of the univariate and multivariate analyses of risk factors for mortality are shown in Table 4. Univariate analysis revealed significant difference between the survivor and nonsurvivor subgroups, with a significantly higher percentage of the nonsurvivors being older, in the ICU on mechanical ventilation and CVP line, and received either corticosteroid, carbapenem, or tigecycline antibiotics. In addition, significantly more of the nonsurvivors had more comorbidities. However, no difference was observed in the CCI between those that survived and those who did not (*p* = 0.857). The control subjects had more chances of survival as shown by the Kaplan-Meier curve *p* = 0.013 (Figure 1). Cumulative survival time was shorter among the cases than controls ((22.4% versus 80.6%) OR 3.28 (0.95–11.55)). None of these variables, however, showed any significance when subjected to multivariable analysis.

The majority of the cases were treated with a colistin-based regimen (55%), either alone (68.8%) or in combination with tigecycline (31.2%). Among those who received any colistin-based regimen, 50% died, segregated by colistin only (45.5%) and colistin-tigecycline combination (60%). Other antibiotics used in the treatment of the CRE included ciprofloxacin (24%), tigecycline (10.5%), amikacin (6.9%), and nitrofurantoin (3.6%).

## 4. Discussion

Carbapenem resistance among the Enterobacteriaceae is an emerging phenomenon of vast clinical and public health importance. This study was necessitated by an observation of a rising trend of infections due to multidrug resistant Gram-negative (MDR GN) pathogens in our institution [15]. Recent studies from other Middle Eastern countries also revealed reduced susceptibility of* K. pneumoniae* to carbapenems [22, 23]. However, this is the first case-control study addressing this topic among Enterobacteriaceae from the region.

Antibiotic resistance among hospitalized patients remains a major global public health problem with attendant increase in healthcare costs, as well as treatment failures, in addition to mortality [2, 10–12]. Carbapenems are often the antibiotics of last resort to treat infections due to extended spectrum *β*-lactamase- (ESBL) or plasmid-mediated AmpC- (pAmpC-) producing organisms. These pathogens are frequently also resistant to other antibiotic classes including quinolones, aminoglycosides, and trimethoprim-sulfamethoxazole [10–12]. The increasing prevalence of CRE, especially during the past 10 years, has seriously compromised the therapeutic armamentarium against infections due to these organisms [2].

Previous studies reported similar risk factors for carbapenem resistant* K. pneumoniae* infection and demonstrated associations with length of hospital stay, ICU admission, use of CVC, recent solid-organ or stem-cell transplantation, receipt of mechanical ventilation, and exposure to broad-spectrum antibiotics [3, 7, 8]. Variables found to be associated with CRE infection in our study include the use of urinary catheter, mechanical ventilation, dialysis in addition to ICU stay, and surgery. Among antibiotics, prior exposure to piperacillin-tazobactam, carbapenem, quinolone, and metronidazole was associated with infection with CRE. Contrary to our finding, Falagas et al. [8] reported that exposure to anti-pseudomonal antibiotics was not associated with CRE infection. Not being in the ICU is protective against CRE infection. These findings are in agreement with previous studies [24–26]. Ho et al. [27] however reported no significant increase in the rate of carbapenem resistance among their patients during the period 2006 to 2010 despite a significant increase in consumption. Recent studies from Saudi Arabia [28] and other Gulf countries [29] showed that OXA-48 and NDM-1 are the dominant carbapenemases among Enterobacteriaceae with low prevalence of VIM.* Klebsiella pneumoniae* accounts for the largest proportion of infections among our cases and controls (51.7% versus 53.4%, resp.), with the least common isolate being* Citrobacter* spp. (3.5% in both groups). This was corroborated by a recent study from Asian countries between 2000 and 2012 [30] where* K. pneumoniae* was reported to be the leading cause of nosocomial infections (39.3%), while* Citrobacter* spp. caused the least (4.5%).

The optimal treatment of infection due to CRE is uncertain, and antibiotic options are limited. The presence of a KPC or metalloenzyme carbapenemase confers resistance to all commonly used antibiotics [31]; hence antimicrobial susceptibility results for agents outside the *β*-lactam and carbapenem classes guide the selection of antibiotic therapy in such cases. This calls for the expansion of the antibiograms used in most centers, which ordinarily do not include agents like colistin or polymyxin B, tigecycline, aztreonam, and fosfomycin (especially for urinary isolates) [32]. The use of two or more antimicrobial agents in combination for the treatment of CRE has been in practice despite limited clinical data because of the high mortality associated with monotherapy. Clinical evidence suggests that treatment with combination therapy may improve outcome [33–38]. Significantly more treatment failures were reported among cases treated with monotherapy compared with combination as published in a recent review of 38 articles that included case reports and case series (49% versus 25%, *p* = 0.01) [39]. In a retrospective review of 125 patients with bacteremia due to KPC gene-harboring* K. pneumoniae* the overall mortality rate at 30 days was 42% [35]. The mortality rate was lower among patients who received combination therapy with two or more drugs (27/79 (34%)) compared with monotherapy with colistin, tigecycline, or gentamicin (25/46 (54%)). Patients treated with a combination of a polymyxin plus tigecycline had a mortality rate of 30 percent (7 of 23), while the regimen of colistin, tigecycline, and extended-infusion* meropenem* (a dose of 2 grams infused over three or more hours every eight hours) was associated with the lowest mortality rate (2 of 16 (12.5%)). Colistin, tigecycline, aminoglycosides, and carbapenems were considered in the prescribed regimen even if the antibiogram showed resistant isolates.

A systematic review of 20 observational studies also concluded that combination therapy may offer a survival advantage in severely ill patients [38]. Colistin formed the backbone of the treatment received by our patients, with 55% receiving a colistin-based regimen. Out of these, 68.8% were treated with colistin only, while 31.2% received a combination of colistin and tigecycline, whereas ciprofloxacin (24.2%), tigecycline (10.3%), amikacin (6.9%), and nitrofurantoin (3.6%) were some of the monotherapy regimens in our cohort. These encouraging results from the use of combination therapy have made physicians, mainly out of desperation, adopt the practice as standard of care in the treatment of CRE infections.

We found a significantly higher mortality rate among those with CRE infection (*p* = 0.031). From a recent review by Falagas et al. [38] the use of combination antimicrobials like colistin with tigecycline, colistin with carbapenem, and tigecycline with gentamicin might result in lower mortality than with other combinations of antibiotics, although this ranged between 50% and 60% for patients in critical care and non-ICU settings, respectively. This figure is similar to ours where mortality was 50% among those who received colistin either alone or in combination. Recently, Vardakas et al. [39] showed that the triple combination of tigecycline, colistin, and an aminoglycoside in the treatment of CRKP had lower mortality (2/8, 25%) compared with any other combination (*p* < 0.002). With the increased use of colistin and tigecycline to treat infections due to CRE, resistance to these lifesaving medications has emerged [40–43].

## 5. Limitations

Our study has limitations. In addition to the relatively small number of patients, matching control patients to cases was a challenge due to the rarity of some of the organisms isolated. Being a single-center study, these results may not be generalizable to other centers where different factors might be contributing to similar infections. However, we thought that we should share our experience with these emerging and increasingly resistant organisms and assess the likely factors that could be contributory to the acquisition of infections due to CRE and describe their treatment and outcome.

## 6. Conclusions

From this single-center observational case-control study we have identified duration of index admission, prior antibiotic use, ICU stay, and invasive procedures to be independently associated with CRE infection. Infection with CRE was also associated with higher mortality compared with CSE. Available evidence from nonrandomized studies, as well as from our limited experience at our center, suggests that combination antibiotic treatment may offer a comparative survival advantage over monotherapy. Improved hand hygiene, barrier nursing, continuous education, minimizing device use, enhanced surveillance, and antimicrobial stewardship will limit CRE transmission in healthcare facilities. Well-designed randomized controlled trials are required to address this crucial question of everyday clinical practice as we await the development of novel antimicrobial agents with reliable efficacy against MDR GNR.

## Figures and Tables

**Table 1:** Baseline demographic and clinical characteristics among the study population.

Variables	Cases (*n* = 29)	Controls (*n* = 58)
Mean age years ± SD (range)	55.4 ± 3.8 (17–85)	54.7 ± 2.6 (15–94)
Gender		
Male	18 (62.1%)	32 (55.2%)
Female	11 (37.9%)	26 (44.8%)
Admission unit		
Medical	9 (31.0%)	26 (44.8%)
Surgical	7 (24.1%)	9 (15.5%)
Intensive care	13 (44.8%)	23 (39.7%)
Source of patient		
Home	22 (75.9%)	47 (81.0%)
Hospital	7 (24.1%)	11 (19.0%)
Sites of infection		
Bloodstream	4 (10.3%)	7 (12.1%)
Skin and soft tissue	7 (24.1%)	10 (17.2%)
Urinary tract	7 (27.6%)	17 (29.3%)
Body fluid	11 (38%)	24 (41.4%)
Infecting organisms		
*Klebsiella pneumoniae*	15 (51.7%)	31 (53.4%)
*Escherichia coli*	7 (24.1%)	13 (22.4%)
*Enterobacter *sp.	6 (20.7%)	12 (20.7%)
*Citrobacter *sp.	1 (3.5%)	2 (3.5%)
Duration of prior hospitalization in the past 3 months (days) ± SD (range)	5.1 ± 1.8 (0–31)	5.1 ± 1.8 (0–31)
Duration of current hospitalization (days) ± SD (range)	56.1 ± 16.7 (0–421)	26.0 ± 5.2 (0–180)
Total number of comorbidities ± SD (range)	3.3 ± 0.3 (1–6)	2.2 ± 0.2 (0–6)
Number of procedures	5.0 ± 0.3 (1–8)	2.8 ± 0.3 (0–7)
Carlson comorbidity index	3.1 ± 0.5 (0–12)	1.9 ± 0.3 (0–7)
Mortality	9 (31.0%)	7 (12.1%)

**Table 2:** Univariate analysis of risk factors associated with CRE and CSE infections.

Characteristics	CRE (*n* = 29)	CSE (*n* = 58)	Univariate OR (95% CI)	*p* value
Female sex	11 (37.9%)	26 (44.8)	0.75 (0.30–1.87)	0.540
Comorbid conditions				
Diabetes mellitus	18 (62.1%)	28 (48.3%)	1.75 (0.71–4.36)	0.224
Pulmonary disease	5 (17.2)	13 (22.4%)	0.72 (0.23–2.26)	0.574
Renal disease	14 (48.3%)	13 (22.4%)	3.23 (1.24–8.39)	0.014
Liver disease	5 (17.2%)	4 (6.9%)	2.81 (0.69–11.4%)	0.153
Cardiovascular disease	19 (656.5%)	27 (46.6%)	2.18 (0.87–5.49)	0.095
Malignancy	10 (34.5%)	13 (22.4%)	1.82 (0.61–4.87)	0.229
Neurologic disease	13 (44.8%)	20 (34.5%)	1.54 (0.62–3.84)	0.349
Clinical characteristics				
CVC placement	18 (62.1%)	26 (44.8%)	2.01 (0.81–5.01)	0.129
Urinary catheter	26 (89.7%)	33 (56.9%)	6.57 (1.78–24.17)	0.002
ICU stay	22 (75.9%)	28 (48.3%)	3.36 (1.25–9.10)	0.014
Surgery	23 (79.3%)	31 (53.4%)	3.34 (1.18–9.41)	0.019
Mechanical ventilation	19 (65.5%)	20 (34.5%)	3.61 (1.41–9.22)	0.006
Dialysis	8 (27.6%)	4 (6.9%)	5.14 (1.40–18.90)	0.017
Prior antibiotic use				
Use of any antibiotic	18 (62.1%)	11 (19%)	6.99 (2.58–18.94)	<0.001
Vancomycin	4 (13.8%)	3 (5.2%)	2.93 (0.61–14.10)	0.215
Piperacillin-tazobactam	12 (41.4%)	0 (0%)	81.9 (5.73–46470974.82)	<0.01
Carbapenem	9 (31%)	3 (5.2%)	8.25 (2.03–33.57)	0.002
Cephalosporin				
First generation	2 (6.9%)	5 (8.6%)	0.78 (0.14–4.31)	1.000
Second generation	3 (10.3%)	1 (1.7%)	6.58 (0.65–66.27)	0.106
Third generation	2 (5.1%)	3 (5.1%)	2.04 (0.12–33.76)	1.000
Fourth generation	2 (6.9%)	0 (0%)	8.59 (0.36–5786949.94)	0.109
Quinolone	9 (31%)	2 (3.4%)	12.60 (2.51–63.35)	<0.01
Metronidazole	8 (27.6%)	1 (1.7%)	21.71 (2.56–184.23)	<0.01
Colistin	1 (3.4%)	0 (0%)	4.14 (0.07–3345908.39)	0.333

**Table 3:** Multivariable analysis for the risk factors associated with CRE infection.

Variable	OR	95% CI	*p* value
Duration of current hospitalization (days)	1.014	1.003–1.025	**0.015**
Number of procedures	3.997	1.679–9.516	**0.002**
Carbapenem use	20.403	1.769–235.379	**0.016**
Not being in the ICU	0.027	0.001–0.496	**0.015**

**Table 4:** Univariate analysis of factors associated with 30-day mortality.

Variable	Nonsurvivors (*n* = 16)	Survivors (*n* = 71)	OR (95% CI)	*p* value
Age	66.9 ± 4.1 (37, 89)	52.2 ± 2.3 (15, 94)	—	0.007
Gender			3.98 (0.94–19.40)	0.064
Male	13 (26.0)	37 (74.0)		
Female	3 (8.1)	34 (91.9)		
Admission site			9.04 (2.10–44.77)	<0.001
ICU	13 (36.1)	23 (63.9)		
Medical	1 (2.9)	34 (97.1)		
Surgical	2 (12.5)	14 (87.5)		
Pulmonary disease			8.86 (2.28–35.86)	<0.001
No	7 (10.1)	62 (89.9)		
Yes	9 (50.0)	9 (50.0)		
Renal disease			3.78 (1.08–13.49)	0.034
No	7 (11.7)	53 (88.3)		
Yes	9 (33.3)	18 (66.7)		
Immunotherapy			5.45 (1.46–20.78)	0.007
No	8 (11.8)	60 (88.2)		
Yes	8 (42.1)	11 (57.9)		
CVP line			49.14 (3.64–27367830.40)	<0.001
No	0 (.0)	43 (100.0)		
Yes	16 (36.4)	28 (63.6)		
Foley catheter			7.28 (1.35–51.82)	0.015
No	2 (7.1)	26 (92.9)		
Yes	14 (23.7)	45 (76.3)		
Stayed in ICU			34.82 (2.59–19367353.0)	<0.001
No	0 (.0)	37 (100.0)		
Yes	16 (32.0)	34 (68.0)		
Mechanical ventilation			29.37 (3.65–632.02)	<0.001
No	1 (2.1)	47 (97.9)		
Yes	15 (38.5)	24 (61.5)		
Carbapenem			3.79 (1.08–13.49)	0.034
No	7 (11.7)	53 (88.3)		
Yes	9 (33.3)	18 (66.7)		
Tigecycline			10.30 (1.77–65.67)	0.004
No	11 (13.9)	68 (86.1)		
Yes	5 (62.5)	3 (37.5)		
